# Novel *Borrelia* species detected in echidna ticks, *Bothriocroton concolor*, in Australia

**DOI:** 10.1186/s13071-016-1627-x

**Published:** 2016-06-14

**Authors:** Siew-May Loh, Alexander W. Gofton, Nathan Lo, Amber Gillett, Una M. Ryan, Peter J. Irwin, Charlotte L. Oskam

**Affiliations:** Vector and Water-Borne Pathogen Research Laboratory, School of Veterinary and Life Sciences, Murdoch University, Perth, Western Australia Australia; School of Biological Sciences, The University of Sydney, Sydney, New South Wales Australia; Australia Zoo Wildlife Hospital, Beerwah, Queensland Australia

**Keywords:** Tick-borne disease, *Bothriocroton concolor*, *Borrelia*, Echidna, Australia

## Abstract

**Background:**

To date, little has been documented about microorganisms harboured within Australian native ticks or their pathogenic potential. Recently, a *Borrelia* sp. related to the Relapsing Fever (RF) group was identified in a single tick removed from a wild echidna (*Tachyglossus aculeatus*). The present study investigated the presence of *Borrelia* in 97 *Bothriocroton concolor* ticks parasitizing echidnas in Queensland, New South Wales, and Victoria, Australia, using nested PCR with *Borrelia*-specific primers targeting the 16S rRNA (16S) and *flaB* genes.

**Results:**

*Borrelia*-specific PCR assays confirmed the presence of a novel *Borrelia* sp. related to the RF and reptile-associated (REP) spirochaetes in 38 (39 %) *B. concolor* ticks. This novel *Borrelia* sp. was identified in 41 % of the *B. concolor* ticks in Queensland and New South Wales, but not in any ticks from Victoria. The resulting *flaB* sequences (407 bp) were 88 and 86 % similar to the *flaB* sequences from *Borrelia turcica* and *Borrelia hermsii*, respectively. Of the ticks confirmed as *Borrelia*-positive following the *flaB* assay, 28 were positive with the 16S assay. Phylogenetic analysis of the 16S sequences (1097 bp) suggests that these sequences belong to a novel *Borrelia* sp., which forms a unique monophyletic clade that is similar to, but distinct from, RF *Borrelia* spp. and REP-associated *Borrelia* spp.

**Conclusions:**

We conclude that the novel *Borrelia* sp. identified in this study does not belong to the *Borrelia burgdorferi* (*sensu lato*) complex, and that the phylogenetic analysis of the partial 16S gene sequences suggests it forms a unique monophyletic cluster in the genus *Borrelia*, potentially forming a fourth major group in this genus associated with monotremes in Australia. However, a thorough molecular characterisation will be required to confirm the phylogenetic position of this unique *Borrelia* sp. The zoonotic potential and pathogenic consequences of this novel *Borrelia* sp. are unknown at the current time.

**Electronic supplementary material:**

The online version of this article (doi:10.1186/s13071-016-1627-x) contains supplementary material, which is available to authorized users.

## Background

Ticks (Acari: Ixodida) transmit the greatest diversity of zoonotic pathogens, including bacteria, protozoa and viruses, of any arthropod and are of major concern to the health and wellbeing of humans, wildlife, livestock and companion animals [1]. Globally, ticks are associated with the transmission of many bacterial pathogens; those of most concern belong predominantly to the genera *Anaplasma* [2], *Borrelia* [3], *Ehrlichia* [4], *Francisella* [5] and *Rickettsia* [6]. In Australia, enzootic ticks that parasitise humans and companion animals also serve as hosts for zoonotic pathogens [7].

In Australia, 70 native and introduced tick species have been described, comprising 56 hard ticks (family Ixodidae: genera *Amblyomma, Bothriocroton* (formerly *Aponomma*), *Haemaphysalis*, *Ixodes*, and *Rhipicephalus*,) and 14 soft ticks (family Argasidae: genera *Argas* and *Ornithodoros*) [8]. Only a few of these ticks are known to transmit pathogens associated with human tick-borne diseases (TBD); currently there are three zoonotic TBDs recognised in Australia associated with native tick species. For example, *Rickettsia australis* and *Rickettsia honei*, the causative agents of Queensland tick typhus and Flinders Island Spotted Fever, respectively, are associated with *Ixodes holocyclus* (Australian paralysis tick), *Bothriocroton hydrosauri* (reptile tick), and *Ixodes tasmani* (marsupial tick) [9–12]. In addition, *Coxiella burnetii*, the causative agent of Q fever, has been detected in *Haemaphysalis humerosa*, the bandicoot tick [13, 14], and *Amblyomma triguttatum*, the ornate kangaroo tick [15]. These ticks are thought to play a role in the life-cycle of *C. burnetii* and this pathogen has been detected in a number of wild animals [16, 17]. Although wildlife are often suggested to be reservoirs for such pathogens in Australia and abroad [16, 18], the importance of wildlife ticks in the ecology of these pathogens is often overlooked or neglected.

Spirochaetes in the genus *Borrelia* are transmitted by arthropods and are classified traditionally into two groups: Lyme Borreliosis (LB) *Borrelia burgdorferi* (*sensu lato*) (*s.l*.), transmitted by several species of ixodid ticks, and Relapsing Fever (RF) *Borrelia*, transmitted primarily by argasid ticks (tick-borne RF, TBRF) [19], a few species by ixodid ticks [20–23], and also by lice [24]. The LB group is the most significant from a human health perspective [25]. The LB group consists of 18 species, of which the principal LB-causing agents include *Borrelia afzelii*, *Borrelia burgdorferi* (*sensu stricto*) (*s.s*.), and *Borrelia garinii*, which are transmitted by the ticks *Ixodes pacificus* and *Ixodes scapularis* in the United States, *Ixodes ricinus* in Europe, and *Ixodes persulcatus* in Europe and Asia [25].

The TBRF *Borrelia* group is conventionally divided geographically into ‘Old World’ RF organisms such as *Borrelia crocidurae*, *Borrelia duttonii* and *Borrelia hispanica*; and ‘New World’ RF species such as *Borrelia hermsii*, *Borrelia parkeri* and *Borrelia turicatae* [26, 27]. Although RF *Borrelia* are predominantly associated with soft ticks, well-known examples of hard ticks-associated TBRF *Borrelia* include: *Borrelia miyamotoi*, isolated from *I. persulcatus* and *I. ricinus* in Europe and Asia [28–30] and *I. scapularis* and *I. pacificus* in north-eastern and western United States, respectively [31, 32]; *Borrelia lonestari* in *Amblyomma americanum* [33]; ‘*Candidatus Borrelia texasensis*’ in *Dermacentor variabilis* in the southern states of North America [34]; and *Borrelia theileri* in *Rhipicephalus* (*Boophilus*) *microplus* [21]. While also recorded in Europe, Asia and North America [35–37], TBRF is a common bacterial infection in several regions in Africa resulting in febrile illness and spirochaetaemia [38–40].

In 2003, a novel *Borrelia* sp. was isolated from a hard tick, *Hyalomma aegyptium*, removed from a tortoise in Istanbul, Turkey [41]. This species was later named *Borrelia turcica* [41, 42] and is genetically distinct from the LB and RF spirochaetes. Further studies have since supported a third major *Borrelia* group classification, designated the reptile-associated (REP) *Borrelia* sp. group [43].

In Australia, three borreliae have been reported: *B. theileri*, the causative agent of bovine spirochaetosis worldwide, transmitted by the cattle tick, *R.* (*Boophilus*) *australis* [44, 45]; *Borrelia anserina* associated with poultry and transmitted by the soft tick, *Argas persicus* [7, 46, 47]; and *Borrelia queenslandica* from long-haired rats, *Rattus villosissimus*, in north-west Queensland, which at the time could not be detected within the proposed tick vector, *Ornithodoros gurneyi* [48]. Spirochaetes have also been observed within blood films of bandicoots, cattle, kangaroo and rodents [49] and in *I. holocyclus* and *Haemaphysalis* spp. ticks, collected from companion animals and livestock [50].

Echidnas, also known as spiny anteaters, are egg-laying mammals classified under the order Monotremata and belonging in the family Tachyglossidae [51]. The short-beaked echidna (*T. aculeatus*) is found in Australia and New Guinea, and is comprised of five subspecies (ssp.): *T. a. acanthion*, *T. a. aculeatus*, *T. a. multiaculeatus*, and *T. a. setosus*, can be found exclusively in Australia, while *T. a. lawesii* is found in New Guinea [51]. In a recent molecular survey of bacteria associated with native Australian human-biting ticks, a novel *Borrelia* sp. related to the RF group was identified in a single *I. holocyclus* tick removed from an echidna host [52]. This finding prompted the current investigation to further assess the occurrence and phylogenetic position of *Borrelia* sp. in ticks collected from echidnas in three regions of Australia and to provide greater insight into its distribution in Australia.

## Methods

### Tick sample collection and identification

A total of 97 ticks were collected from 22 echidnas (*T. aculeatus* ssp.) by veterinarians at the Australian Zoo Wildlife Hospital in Beerwah, Queensland (*n* = 81), wildlife carers at the Wild Days Wildlife shelter in Narre Warren, Victoria (*n* = 4), and through public submission from Wagga Wagga, western New South Wales (*n* = 12). Ticks were preserved in 70 % ethanol immediately after removal and sent to Murdoch University for species identification and molecular analyses. Twenty-six male and 71 female *B. concolor* ticks were identified based on morphological assessment according to the standard keys for identifying Australian ticks [53].

### DNA extraction

Prior to DNA extraction, ticks were surface-sterilised with 10 % sodium hypochlorite and washed with sterile and DNA-free water, and 70 % ethanol. The extractions were carried out as described by Gofton et al. [52]. Negative controls were treated in an identical manner. *Borrelia afzelii* and *B. burgdorferi* (*s.s*.) DNA previously extracted from questing nymphal *I. ricinus* ticks (LN1, LN6, LN7 and LN9) from Leipzig, Germany [52], were used as positive controls in all PCR assays. DNA from one *Borrelia* sp.-infected female *I. holocyclus* tick (NL230) described in Gofton et al. [52], collected from an echidna host in New South Wales was also reanalysed in the present study.

### *Borrelia*-specific PCR and sequencing

To determine the presence of *Borrelia* sp. within the 97 *B. concolor* ticks, and one *I. holocyclus* tick, DNA extractions were subjected to two *Borrelia* genus-specific PCR assays. *Borrelia*-specific nested-PCR assays were conducted targeting the 16S rRNA (16S) and *flaB* genes (Table 1). Each 25 μl PCR reaction contained 1× PerfectTaq buffer, 2.5 mM MgCl_2_, 1 mM dNTPs, 400 nM of each primer, 1.25 U PerfectTaq polymerase, and 2 μl undiluted DNA. Both the primary and nested *Borrelia* 16S PCR assays were performed with the following thermal conditions: initial denaturation at 95 °C for 5 min, 35 cycles of denaturation at 95 °C for 30 s, annealing at 51 °C for 40 s, and extension at 72 °C for 2 min, and a final extension at 72 °C for 5 min. The *flaB* PCR assays were performed with an initial denaturation at 95 °C for 5 min, 35 cycles of denaturation at 95 °C for 30 s, annealing at 52 °C (primary) or 55 °C (nested) for 30 s, and extension at 72 °C for 30 s, and a final extension at 72 °C for 5 min. No-template controls and positive controls were included in all PCR assays.

**Table 1:** Primers used for *Borrelia*-specific 16S rRNA and *flaB* genes amplification in this study, including primer sequences, annealing temperature and expected product size

Gene	Primer	Sequence (5' – 3')	Annealing temperature	Expected product size (bp)	Reference
16S	External				
	Bor-16 F	TGCGTCTTAAGCATGCAAGT			
	Bor-1360R	GTACAAGGCCCGAGAACGTA	51 °C	1,344	This study
	Internal				
	Bor-27 F	CATGCAAGTCAAACGGAATG			
	Bor-1232R	ACTGTTTCGCTTCGCTTTGT	51 °C	1,205	This study
*flaB*	External				
	FlaB280F	GCAGTTCARTCAGGTAACGG			
	FlaRL	GCAATCATAGCCATTGCAGATTGT	52 °C	645	[33, 66]
	Internal				
	flaB_737F	GCATCAACTGTRGTTGTAACATTAACAGG			
	FlaLL	ACATATTCAGATGCAGACAGAGGT	55 °C	407	[33, 66]

Amplified PCR products were electrophoresed through 1–2 % agarose gels, stained with GelRed (Biotium), and visualised under UV light. Amplicons of expected sizes were excised from the gel and purified with the Wizard® SV Gel and PCR Clean-Up System (Promega Madison, WI, USA,), according to the manufacturer’s recommendations. Purified PCR products were sequenced with both forward and reverse nested PCR primers using BigDye v3.1 terminator on an ABI 373096 Capillary Sequencer (Life Technologies, USA).

### Sequence analysis

Trimmed 16S sequences (1097 bp) generated in this study, together with sequences from other *Borrelia* spp. retrieved from GenBank, were aligned using MAFFT v7.017 [54], and the alignment was refined using MUSCLE [55]. *Spirochaeta americana* ASpG1 strain (AF373921) [56] was used as an outgroup. Following multiple sequence alignments, *MEGA* version 6 [57] was used to determine the most suitable nucleotide substitution model based on the Bayesian Information Criterion (BIC). General time reversible (GTR) model was selected and the *Borrelia* 16S phylogenetic tree was generated using FastTree 2 [58], with 20 rate categories of site and resampling 1000 times. 16S sequences generated in this study were deposited in GenBank under accessions KU954112 to KU954115, and *flaB* sequences were deposited in GenBank under the accession KX192143 to KX192150.

## Results

### Molecular and phylogenetic analyses

A partial fragment (407 bp) of the *Borrelia flaB* gene was successfully amplified in three male and 35 female *B. concolor* ticks (38/97, 39 %) and also in the single *I. holocyclus* tick from the previous study [52] (Table 2). All ticks (*n* = 4) from Victoria were negative for *Borrelia* spp. *Bothriocroton concolor* ticks that were *Borrelia*-positive originated from Queensland and New South Wales (38/93, 41 %) (Additional file 1: Table S1). BLAST analysis showed that, with 100 % query coverage, these *flaB* equences shared 87.4–88.6 % similarity with *flaB* sequences from *B. turcica* (GenBank: AB109245; AB109244; AB109241) [41, 42], and with 85.5–86.4 % similarity with *B. hermsii* (GenBank: AY597795; AY597798) [59]. BLAST analysis of the positive samples isolated from *I. ricinus* ticks revealed 100 % identity with the LB spirochaetes, with samples LN1, LN7 and LN9 identical to *B. afzelii* at the *flaB* locus (GenBank: GU826786) [60]; and LN6 identical to *B. burgdorferi* (GenBank: DQ016620; AF386506; AB035618) [61, 62].

**Table 2:** Summary of the sex and life stages of the tick specimens used in this study, including the positive controls *Ixodes holocyclus* and *I. ricinus*. The geographical regions where the ticks were collected either from a host or vegetation were recorded and the number of ticks positive for *Borrelia* genes is presented in terms of percentage

Ticks	Region	Host/questing	*n*	Gene (% of positive samples)	
				16S	*flaB*
*B. concolor*					
M	QLD	Echidna	12	0	16.6
	NSW	Echidna	10	10	10
	VIC	Echidna	4	0	0
F	QLD	Echidna	69	36.2	50.7
	NSW	Echidna	2	100	100
*I. holocyclus*					
F	NSW	Echidna	1	100	100
*I. ricinus*					
N	Leipzig, Germany	Questing	4	100	100

*Abbreviations*: *M* male adult, *F* female adult, *N* nymph, *QLD* Queensland, *NSW* New South Wales, *VIC* Victoria

The *Borrelia*-specific 16S PCR assay was positive for one male and 27 female *B. concolor* ticks (28/97; 29 %), all of which were also positive at the *flaB* locus (Table 2). Three distinct *Borrelia* 16S sequences were produced from *B. concolor* ticks, which differed by only a single nucleotide polymorphism (SNP) (99.9 % similarity) between the isolates. Alignment with the *Borrelia* sequence isolated from an *I. holocyclus* tick, from the previous study [52], revealed that the sequences shared > 99 % identity, with one verified SNP. The three unique *Borrelia* 16S sequences from *B. concolor* ticks were putatively designated *Borrelia* sp. Aus A, *Borrelia* sp. Aus B, and *Borrelia* sp. Aus C, which occurred in 22, five, and one sample, respectively. The *Borrelia* 16S sequence from the *I. holocyclus* tick was putatively designated *Borrelia* sp. NL230.

**Fig. 1:**
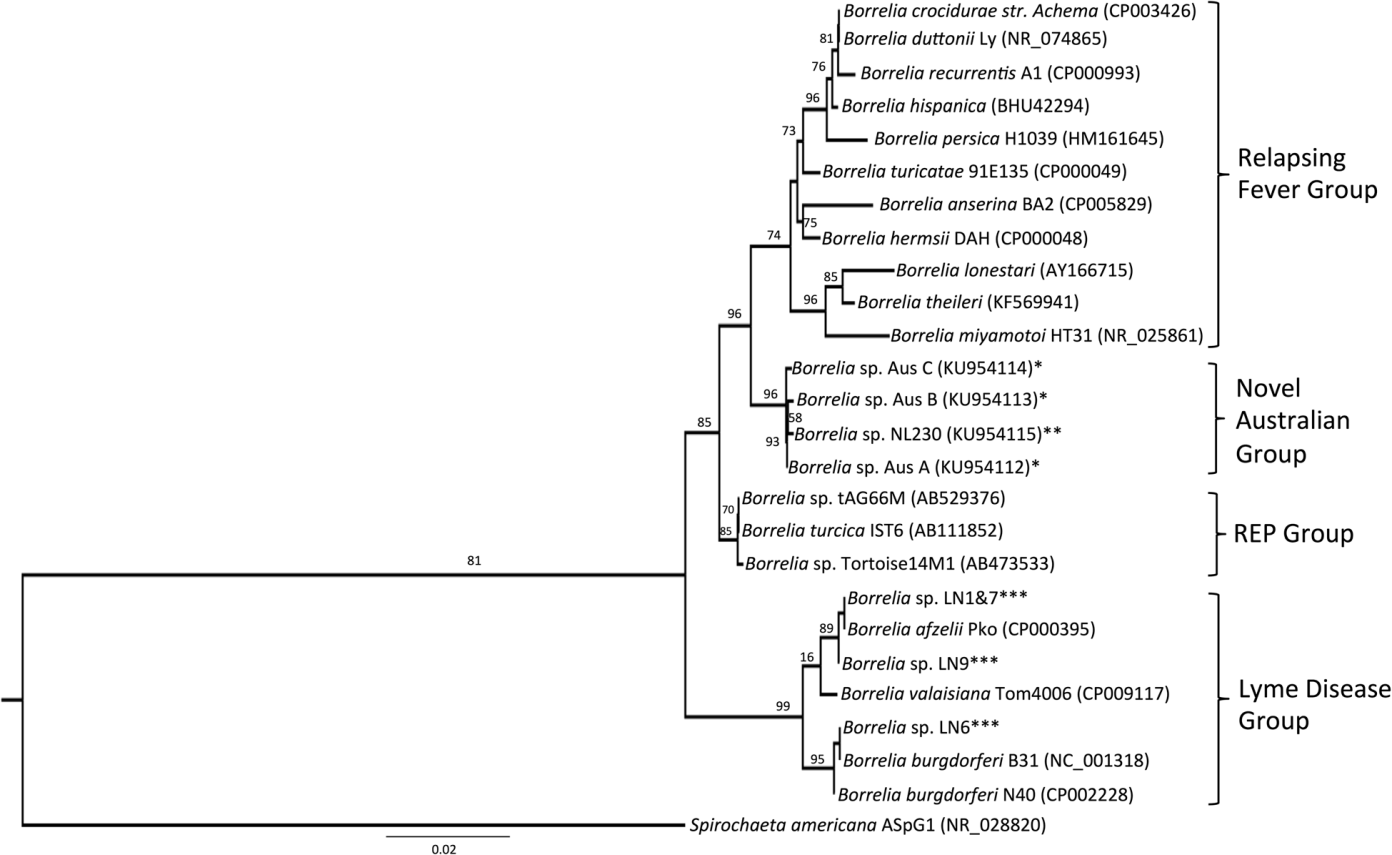
Molecular phylogenetic analysis of 1097 bp fragment of 16S rRNA of the *Borrelia* sp. amplified in *B. concolor* ticks from echidnas. Phylogenetic distances were inferred using FastTree 2 [57], and specimens were compared with the *B. burgdorferi* (*sensu lato*) complex, the TBRF group, and the REP-associated spirochaetes. “*” represents *Borrelia* sequences from *B. concolor* ticks; “**” indicates *Borrelia* sequence from *Ixodes holocyclus* tick; and “***” represents positive controls from *I. ricinus* nymphs

Phylogenetic analysis indicates that *Borrelia* 16S sequences from this study form a unique monophyletic clade, with high confidence, and that is most similar to, but distinct from the RF and REP *Borrelia* groups (Fig. 1). *Borrelia* 16S sequences from these echidna ticks were most dissimilar to the LB group (96.1–96.7 % similarity); and most similar (98.6–98.7 %) to both *Borrelia* sp*.* tAG66M and *B. hermsii* DAH (Additional file 2: Table S2). *Borrelia* 16S sequences generated from nymphal *I. ricinus* ticks clustered within the *B. burgdorferi* (*s.l*.) group, with high (99.9–100 %) similarity to *B. burgdorferi* and *B. afzelii* (Fig. 1).

## Discussion

This study presents the first molecular characterisation in Australia of a novel *Borrelia* sp. identified in *B. concolor* (this study) and in a single *I. holocyclus* tick [52], with a close relationship to the RF and REP-associated *Borrelia* groups. The current investigation aimed to provide further evidence for *Borrelia* in echidna ticks, if any, and to validate the previous findings [52] with an increased sample size and by targeting the highly conserved *Borrelia* housekeeping genes (e.g. *flaB* and 16S rRNA).

The reduced sensitivity of detecting *Borrelia* with the 16S PCR assay, compared to the *flaB* PCR assay, may be explained by the reduced PCR efficiency that is typically observed when amplifying longer gene fragments. For this reason, PCR assays targeting short gene fragments, such as the *flaB* assay used in the present study, are recommended for sensitive detection of *Borrelia* spp. However, short gene fragments are often inadequate to produce meaningful phylogenetic reconstructions, and therefore must be complimented by assays that amplify longer gene sequences, albeit with less sensitivity, such as the 16S assay in the present study.

Recently, REP-associated *Borrelia* spp. isolated from reptile ticks, *H. aegyptium*, were shown to form a monophyletic clade [42], while sharing a common ancestor with the RF and LB *Borrelia* groups, supporting the notion of a third *Borrelia* group [43]. Likewise, the *Borrelia* sp. identified in this study also forms a unique monophyletic clade, and may form a fourth major phylogenetic *Borrelia* group. However, further analyses of other *Borrelia* housekeeping genes, such as *gyrB*, *groEL*, *glpQ* genes, and 23S-16S intergenic spacer region [43], and morphological characterisation are necessary for a complete description of this novel *Borrelia* sp.

The current investigation has provided new records of a *Borrelia* sp. found in a native Australian hard tick, *B. concolor* [44, 45, 52]. Currently, the degree of similarity and differences between the *Borrelia* sp. characterised in the present study and *B. queenslandica*, identified in long-haired rats, *R. villosissimus* [48], is unknown as molecular data does not exist for this species.

All ticks removed from echidna hosts for the present study were *B. concolor*, a known specialist tick species usually restricted to parasitising echidnas (family Tachyglossidae) [53, 63]; however, kangaroos (*Macropus fuliginosus fuliginosus*) were recently recorded as a new host of *B. concolor* on Kangaroo Island, South Australia [64]. *Bothriocroton concolor* has a relatively wide distribution including both coastal and sub-coastal regions of Queensland and New South Wales, as well as inland New South Wales; whereas the distribution of *I. holocyclus* (the host of the first isolate reported by Gofton et al. [52]) is mainly restricted to coastal regions of the eastern Australia [53]. It has been reported that echidnas can also host other tick species such as *Amblyomma australiense*, *Amblyomma echidnae*, *Amblyomma moyi, Amblyomma papuanum*, *Bothriocroton tachyglossi*, *Bothriocroton undatum, Haemaphysalis humerosa* and *I. tasmani* [53]. Therefore, different tick species that feed on the same host may become infected through blood meals, if the echidna serves as a bacteraemic vertebrate reservoir for this bacterium.

Generally, many tick-borne microorganisms circulate within unique sylvatic cycles in an ecosystem, and wildlife and their ticks play important roles as reservoirs and bridging vectors, respectively [65]. In Australia, wildlife and their ticks have long been considered as reservoirs for a number of tick-borne pathogens [10, 16]. In the case of the vector of *Borrelia* in Australia, the soft tick, *O. gurneyi* from long-haired rats, was proposed as a vector of *B. queenslandica*, however, transmission attempts were unsuccessful [48]. Here in the present study, a novel bacterium harboured within *B. concolor* was identified in two tick genera, suggesting that echidnas may be a potential reservoir of this bacterium. However, whether these ticks are able to acquire this bacterium from infected echidna hosts remains to be confirmed through the analysis of echidna blood samples, and until further studies are completed, the vertebrate reservoir of this spirochaete remains to be determined. Likewise, the role of *B. concolor* and *I. holocyclus* ticks as potential bridging vectors of this novel *Borrelia* sp. remains to be assessed via transmission studies. Despite this, the potential and importance of wildlife and their ticks acting as reservoir and vector in maintaining the persistence of this bacterium in the environment cannot be disregarded. The pathogenic consequences (if any) and potential infectivity of this *Borrelia* sp. to animals and humans are unknown at the present time.

## Conclusions

The current study has identified a novel *Borrelia* sp. harboured within echidna ticks. Phylogenetic analysis of the partial 16S sequences showed that this *Borrelia* forms a unique monophyletic clade that is closely related to the RF and REP *Borrelia* groups and is most dissimilar to the LB group. Moreover, this study highlights the significance of studying Australian wildlife ticks. The presence of a novel *Borrelia* sp. in Australia is of significant public health importance and warrants further investigations to better understand the biology, ecology, pathogenicity (if any), and infectivity of this organism to humans, domestic animals, and wildlife.

## Abbreviations

16S, 16S rRNA; BIC, bayesian information criterion; GTR, general time reversible; LB, Lyme Borreliosis; NSW, New South Wales; QLD, Queensland; REP, reptile-associated; RF, relapsing fever; *s.l.*, *sensu lato*; *s.s.*, *sensu stricto*; SNP, single nucleotide polymorphism; TBD, tick-borne disease; TBRF, tick-borne relapsing fever; VIC, Victoria

## Additional file

Additional file 1: Table S1. Summary of echidna hosts, clinic, region, number and sex of ticks used in this study, and the number of positive amplifications at each gene. (PDF 165 kb) Additional file 2: Table S2. 16S rRNA genetic distance matrix showing percentage nucleotide sequence identity between *Borrelia* sp. from this study and other *Borrelia* spp., with the outgroup, *Spirochaeta americana*. (PDF 165 kb)
