# Supplementary material for: Novel Borrelia species detected in echidna ticks, Bothriocroton concolor, in Australia
Source: Parasit Vectors. 2016 Jun 14;9:339. doi: 10.1186/s13071-016-1627-x (PMC4908759; doi:10.1186/s13071-016-1627-x)
Supplement: Additional file 1: Table S1. — Summary of echidna hosts, clinic, region, number and sex of ticks used in this study, and the number of positive amplifications at each gene. (PDF 165 kb) [file 13071_2016_1627_MOESM1_ESM.pdf]

Additional file 1: Table S1. Summary of echidna hosts, clinic, region, number and sex of ticks used in this study, and the number of positive amplifications at each gene.

Host ID	Clinic	Region	Number of ticks	Sex of ticks	16S rRNA	*flaB*
1440	*	Wagga Wagga, NSW	10	M	1	1
1440	*	Wagga Wagga, NSW	2	F	2	2
1268	Australia Zoo Wildlife Hospital	QLD	10	F	6	7
915	Australia Zoo Wildlife Hospital	QLD	7	F	-	-
931	Australia Zoo Wildlife Hospital	QLD	6	F	3	5
476	Australia Zoo Wildlife Hospital	QLD	1	F	-	-
479	Australia Zoo Wildlife Hospital	QLD	2	F	1	1
500	Australia Zoo Wildlife Hospital	QLD	2	F	1	1
504	Australia Zoo Wildlife Hospital	QLD	1	F	1	1
1232	Australia Zoo Wildlife Hospital	QLD	7	F	5	5
1241	Australia Zoo Wildlife Hospital	QLD	10	F	-	2
406	Australia Zoo Wildlife Hospital	QLD	2	F	-	-
1239	Australia Zoo Wildlife Hospital	QLD	1	M	-	1
1241	Australia Zoo Wildlife Hospital	QLD	7	M	-	-
1265	Australia Zoo Wildlife Hospital	QLD	1	M	-	1
876	Wild Days Wildlife Shelter	Narre Warren North, VIC	4	M	-	-
434	Australia Zoo Wildlife Hospital	QLD	3	M	-	-
1250	Australia Zoo Wildlife Hospital	QLD	2	F	1	1
1261	Australia Zoo Wildlife Hospital	QLD	2	F	1	1
1265	Australia Zoo Wildlife Hospital	QLD	3	F	-	1
538	Australia Zoo Wildlife Hospital	QLD	2	F	1	1
549	Australia Zoo Wildlife Hospital	QLD	2	F	1	1
534	Australia Zoo Wildlife Hospital	QLD	2	F	-	-
1230	Australia Zoo Wildlife Hospital	QLD	2	F	1	2
1239	Australia Zoo Wildlife Hospital	QLD	2	F	2	2
1248	Australia Zoo Wildlife Hospital	QLD	4	F	1	2
Total			97		28	38

* Individual submission, no clinic records.
