# Supplementary material for: Novel Borrelia species detected in echidna ticks, Bothriocroton concolor, in Australia
Source: Parasit Vectors. 2016 Jun 14;9:339. doi: 10.1186/s13071-016-1627-x (PMC4908759; doi:10.1186/s13071-016-1627-x)
Supplement: Additional file 2: Table S2. — 16S rRNA genetic distance matrix showing percentage nucleotide sequence identity between Borrelia sp. from this study and other Borrelia spp., with the outgroup, Spirochaeta americana. (PDF 165 kb) [file 13071_2016_1627_MOESM2_ESM.pdf]

Additional file 2: Table S2. 16S rRNA genetic distance matrix showing percentage nucleotide sequence identity between *Borrelia* sp. from this study and other *Borrelia* spp., with the outgroup, *Spirochaeta americana*.

	**1**	**2**	**3**	**4**	**5**	**6**	**7**	**8**	**9**	**10**	**11**	**12**	**13**	**14**	**15**	**16**	**17**	**18**	**19**	**20**	**21**	**22**	**23**	**24**	**25**
**1. *Borrelia* sp. Aus A**																									
**2. *Borrelia* sp. Aus B**	99.9																								
**3. *Borrelia* sp. Aus C**	99.9	99.8																							
**4. *Borrelia* sp. NL230**	99.9	99.8	99.8																						
**5. *Borrelia* sp. tAG66M**	98.7	98.6	98.6	98.6																					
**6. *Borrelia* sp. Tortoise14M1**	98.6	98.5	98.5	98.5	99.9																				
**7. *Borrelia turcica* IST6**	98.6	98.5	98.5	98.5	99.9	99.8																			
**8. *Borrelia anserina* BA2**	97.5	97.4	97.4	97.4	97.9	98.0	97.8																		
**9. *Borrelia crocidurae* str. Achema**	98.3	98.2	98.2	98.2	98.1	98.2	98.0	98.5																	
**10. *Borrelia duttonii* Ly**	98.3	98.2	98.2	98.2	98.1	98.2	98.0	98.5	100.0																
**11. *Borrelia recurrentis* A1**	98.0	97.9	97.9	97.9	97.8	97.9	97.7	98.3	99.7	99.7															
**12. *Borrelia persica* H1039**	98.0	97.9	97.9	97.9	97.8	97.7	97.7	98.1	99.2	99.2	98.9														
**13. *Borrelia hermsii* DAH**	98.7	98.6	98.6	98.6	98.4	98.5	98.3	98.6	99.0	99.0	98.7	98.5													
**14. *Borrelia turicatae* 91E135**	98.5	98.5	98.5	98.5	98.2	98.3	98.1	98.6	99.2	99.2	98.9	98.7	99.3												
**15. *Borrelia hispanica* BHU42294**	98.2	98.1	98.1	98.1	98.0	98.1	98.1	98.5	99.7	99.7	99.5	99.1	98.9	99.1											
**16. *Borrelia lonestari* AY166715**	97.5	97.4	97.4	97.4	97.0	97.1	96.9	97.3	98.0	98.0	97.7	97.4	97.9	98.3	98.0										
**17. *Borrelia theileri* KAT**	98.2	98.1	98.1	98.1	97.6	97.7	97.5	97.9	98.5	98.5	98.2	97.8	98.5	98.7	98.4	99.0									
**18. *Borrelia miyamotoi* HT31**	97.4	97.4	97.4	97.4	97.1	97.2	97.0	97.7	97.7	97.7	97.4	97.4	98.0	98.0	97.6	98.1	98.5								
**19. *Borrelia* sp. LN1&7**	96.2	96.1	96.3	96.1	96.8	96.7	96.7	96.2	95.9	95.9	95.6	96.3	96.2	96.2	95.9	95.4	95.8	95.6							
**20. *Borrelia afzelii* PKo**	96.2	96.1	96.3	96.1	96.8	96.7	96.7	96.2	95.9	95.9	95.6	96.3	96.2	96.2	95.9	95.4	95.8	95.6	100.0						
**21. *Borrelia* sp. LN9**	96.3	96.2	96.4	96.2	96.9	96.8	96.8	96.3	96.0	96.0	95.7	96.4	96.3	96.3	96.0	95.4	95.9	95.7	99.9	99.9					
**22. *Borrelia valaisiana* Tom4006**	96.6	96.5	96.7	96.5	97.1	97.0	97.0	96.1	96.0	96.0	95.7	96.4	96.4	96.4	96.0	95.6	96.1	95.7	99.4	99.4	99.5				
**23. *Borrelia* sp. LN6**	96.4	96.3	96.4	96.3	96.9	96.8	96.8	95.9	96.2	96.2	95.9	96.4	96.4	96.4	96.2	95.5	96.0	95.4	98.9	98.9	99.0	98.9			
**24. *Borrelia burgdorferi* B31**	96.4	96.3	96.4	96.3	96.9	96.8	96.8	95.9	96.2	96.2	95.9	96.4	96.4	96.4	96.2	95.5	96.0	95.4	98.9	98.9	99.0	98.9	100.0		
**25. *Borrelia burgdorferi* N40**	96.4	96.4	96.5	96.4	97.0	96.9	96.9	96.0	96.1	96.1	95.8	96.3	96.4	96.4	96.1	95.6	96.1	95.5	99.0	99.0	99.1	99.0	99.9	99.9	
**26. *Spirochaeta americana* ASpG1**	81.3	81.4	81.4	81.2	81.6	81.5	81.6	81.2	81.3	81.3	81.0	81.3	81.6	81.7	81.1	80.8	81.0	80.9	80.4	80.4	80.5	81.0	80.4	80.4	80.4
